# Impaired cognition is related to microstructural integrity in relapsing remitting multiple sclerosis

**DOI:** 10.1002/acn3.51100

**Published:** 2020-06-09

**Authors:** Lin Zhao, Angel Ng, Qianyun Chen, Bonnie Lam, Jill Abrigo, Cheryl Au, Vincent C. T. Mok, Adrian Wong, Alexander Y. Lau

**Affiliations:** ^1^ Department of Medicine and Therapeutics Prince of Wales Hospital The Chinese University of Hong Kong Hong Kong SAR China; ^2^ Department of Imaging and Interventional Radiology Prince of Wales Hospital The Chinese University of Hong Kong Hong Kong SAR China

**Keywords:** multiple sclerosis, cognition impairment, magnetic resonance imaging, DTI, Chinese

## Abstract

**Background:**

Cognitive impairment is common in multiple sclerosis (MS). However, the relationship between cognitive deficits and microstructural abnormalities in Chinese MS patients remains unclear. We aimed to investigate the importance of microstructural abnormalities and the associations with cognitive impairment in Chinese MS patients.

**Methods:**

Three‐dimensional T1‐weighted magnetic resonance imaging (MRI) scans were obtained from 36 relapsing remitting MS patients. Diffusion tensor imaging (DTI) scans were acquired for 29 (81%) patients. Cognitive impairment was assessed using a comprehensive neuropsychological battery. Patients were classified into cognitively impaired (CI) group and cognitively preserved (CP) group. Using volBrain and FSL software, we assessed white matter lesion burden, white matter (WM) and gray matter (GM) volumetric as well as microstructural diffusivity. MRI variables explaining cognitive impairment were analyzed.

**Results:**

Fifteen (42%) patients were classified as CI. Verbal learning and memory was the most commonly impaired domain (n = 16, 44%). CI patients had lower mean skeleton fractional anisotropy (FA) value than CP patients (275.45 vs. 283.61 × 10^−3^, *P* = 0.023). The final predicting model including demographic variables and global skeleton mean diffusivity (MD) explained 43.6% of variance of the presence of cognitive impairment (β = 0.131, *P* = 0.041). CI patients showed a widespread change of microstructural integrity comparing to CP patients, which was rarely overlapping with lesion probability map. Microstructural abnormalities in corpus callosum were associated with performance in verbal learning and memory, processing speed and selective attention (*P* < 0.05).

**Conclusion:**

Loss of microstructural integrity demonstrated by DTI helps explain cognitive dysfunction in Chinese MS patients.

## Introduction

Cognitive impairment in multiple sclerosis has been recognized for a long time and occurs in 40‐70% of multiple sclerosis (MS) patients,^1^, ^2^ most commonly affecting memory, information processing speed, attention, and executive functions.^3^, ^4^ Cognitive deficits can happen in early stage of disease, and severely impact patients’ quality of life.^5^ Traditional treatment goals, including reduction of relapses, magnetic resonance imaging (MRI) lesions and prevention of disability worsening, put emphasis mainly on inflammatory activity. Often, ongoing neurodegeneration which accounts for cognitive impairment is overlooked.^6^, ^7^ There are limited options at present to treat cognitive impairment, as its pathogenesis is poorly understood. A sensitive biomarker to detect risk of cognitive dysfunction and treatment‐related cognition improvement is therefore urgently required.^8^

MRI is a powerful non‐invasive diagnostic and monitoring tool for MS and has great potential to detect cognitive dysfunction. However, recent studies showed only weak associations between traditional MRI measures, such as white matter lesion (WML) load and whole brain atrophy, and cognitive performance.^9^ The development of advanced MRI techniques may allow early detection of cognitive dysfunction.^8^, ^10^ White matter (WM), gray matter (GM) and WML were found to account for cognitive dysfunction but the contributions were inconsistent among different studies.^1^ Thalamic atrophy, among the subcortical gray matter structures, is most promising to predict cognitive dysfunction.^10^ Diffusion tensor imaging (DTI) allows for quantitative measurements of the WM microstructural integrity^11^; certain white matter tracts, such as the corpus callosum, cingulum, posterior thalamic tract, show reduced mean skeleton fractional anisotropy (FA) value and increased mean skeleton mean diffusivity (MD) value, and these abnormalities are related to impairment on cognition.^12^, ^13^

Due to the lower prevalence and different genetic background as compared to Caucasian patients, the relationship between cognitive function and advanced MRI parameters in Chinese patients may differ from those reported in Caucasian patients.^14^, ^15^ In this study, we aimed to identify the influence of microstructural integrity, WM atrophy, GM atrophy, thalamic atrophy, WML load and demographic factors on cognitive performance in Chinese relapsing remitting multiple sclerosis (RRMS) patients. Furthermore, we tested the hypothesis that the extent of loss of microstructural integrity differs between patients with preserved cognition and impaired cognition.

## Methods

### Participants

We recruited RRMS patients fulfilling the McDonald criteria 2017, who were free of clinical relapse for at least 3 months, from The Chinese University of Hong Kong ‐ Multiple Sclerosis Registry (CUMSR).^16^ Expanded Disability Status Scale (EDSS) was performed within 1 month of MRI scanning. Cognitive assessment was performed within 3 months on average of MRI scanning. We excluded participants with other psychiatric co‐morbidities. All participants gave written informed consent. The study was approved by Joint Chinese University of Hong Kong – New Territories East Cluster Clinical Research Ethics Committee (Reference number: CRE‐2013.155&CRE‐2014.130).

### MRI acquisition

All MRI scans were performed on a single 3 Tesla scanner (Philips Achieva TX, Best, The Netherlands) using an 8‐channel head coil. Structural imaging included volumetric T1‐weighted and FLAIR sequences of the brain and T2‐weighted sequences of the spinal cord which were all acquired in the sagittal plane. Their parameters were as follows (TR: repetition time, TE: echo time, TI: inversion time, FOV: field of view): (1) Three‐dimensional (3D) T1‐weighted Fast Field Echo (FFE): TR 7.5 ms, TE 3.5 ms, FOV 250 mm, matrix 228 × 208, slice thickness 1.1 mm; (2) 3D T2‐weighted FLAIR with fat suppression —TR 8000 ms/ TI 2400 ms, TE 341 ms, FOV 230 mm, matrix 208 × 208, slice thickness 1.1 mm; (3) T2‐weighted: TR 3666 ms, TE 120 ms, FOV 270 mm, matrix 340 × 254, slice thickness 2mm.

DTI of the whole‐brain was acquired using a single shot echo‐planar imaging diffusion‐weighted sequence (TR = 9060 ms, TE = 60 ms, FOV = 224 mm, matrix 112 × 112, 70 axial slices with an isotropic 2 mm resolution) with 32 gradient directions with non‐collinear diffusion gradients (b‐value of 1000 seconds/mm^2^) and one volume without diffusion weighting (b‐value of 0 seconds/mm^2^).

### Image processing and analysis

*Normalized brain volumes*. Normalized white matter volume (NWMV), normalized gray matter volume (NGMV) and normalized deep gray matter volume (NDGMV) were obtained by using volBrain, an automated MRI brain volumetry system (https://volbrain.upv.es/).^17^ The measured deep gray matter structures included caudate, putamen, thalamus, globus pallidus, hippocampus, amygdala, and accumbens.

#### WML distribution, lesion mask and group lesion probability map (LPM)

WML segmentation was done by lesion growth algorithm (LGA) in lesion segmentation tool (LST),^18^ which is an open source toolbox for Statistical Parametric Mapping (SPM) 12.0.^19^ LGA requires T1‐weighted images in addition to the corresponding FLAIR images. After a fully automatic processing by LST, the lesion mask for every patient was obtained. The lesion masks were double checked by a neurologist. We extracted brain from 3D T1‐weighted images using BET tool in FMRIB Software Library (FSL) v6.0 (www.fmrib.ox.ac.uk/fsl/).^20^ The brain‐ extracted T1‐weighted images were registered to Montreal Neurological Institute (MNI152) 1mm standard space images with 12 DOF affine registration implemented in FLIRT and were refined by non‐linear registration implemented in FNIRT in FSL.^21^, ^22^ A threshold of 0.5 was applied to binarize native space masks. Binary lesion masks were then transformed to standard space by using the transformation matrices and warp fields from the above‐mentioned registrations. Finally, for the group, lesion probability map (LPM) was generated by first merging and then averaging all the standard‐space lesion masks. For each map, voxel intensity represents the frequency of lesion occurrence in that voxel or, in other words, the probability of that voxel being lesion for the group. The lesions were saved when at least 5% patients had the lesion, otherwise were removed from the map. An experienced neurologist carefully visually checked the native FLAIR images to record the distributions of WML.

#### Tract‐Based Spatial Statistics (TBSS)

Eddy was used to pre‐process raw DTI images in order to correct for distortions due to the gradient directions applied.^23^ Subsequently, DTIFIT was used to fit a diffusion tensor model at each voxel and generate FA and MD images. FA maps were fed into the Tract‐Based Spatial Statistic (TBSS) tool.^24^ The FA maps of all patients were aligned into a 1 × 1 × 1mm standard space called FMRIB58_FA by non‐linear registration and averaged to obtain a mean FA skeleton. Finally, each patient’s aligned FA data were projected onto this skeleton. Similar processes were applied to MD maps using the individual registration and projection vectors obtained in the FA nonlinear registration and skeletonization stages. A voxel‐wise cross‐subject statistical analysis was then performed to compare DTI metrics of CI and CP patients. General linear model (GLM) was used to perform group comparison, adjusted for patient’s age, gender and educational years, and applied to the spatial maps using permutation‐based non‐parametric testing (5000 permutations) with correction for multiple comparisons, by using a cluster‐based correction approach and FWE‐corrected *P*‐value < 0.05. GLM was also applied to identify the correlation between DTI metrics and the clinical scores: clinical variables were entered in a correlation analysis as variables of interest, adjusted for the patients’ age, gender and educational years. The resulting statistical maps threshold was set at *P* < 0.05. We further corrected TBSS results using Bonferroni method to avoid inflated type I error and establish the most correlated WM tracts.^25^ Significant WM tracts were localized using FSL WM Atlas.

### Cognitive assessments

Seven cognitive functions were examined. The following cognitive assessments were performed for all subjects: (1) Verbal learning and memory—assessed with The Chinese Auditory Verbal Learning Test (CAVLT), which is a measure of short‐term and long‐term auditory verbal memory. It consists of a 15‐word list with five learning trials, one interruptive trial, one immediate‐recall trial, one delayed‐recall trial, and one recognition trial, respectively. From learning trial 1 to 5, subjects were asked to recall the words after administrator has verbally presented to them. The interruptive trial was performed right after the learning trials. A different word list was presented to the subject in this trial and they were required to recall as many words as they could. In immediate‐recall trial, subjects were asked to recall the words from the learning trial immediately after the interruptive trial, which assesses the short‐term auditory verbal memory. Delayed‐recall trial would be performed after a 30‐min delay^26^; (2) Processing speed—assessed with Trail Making Test (TMT) A, which requires participants to draw lines to connect the numbers in ascending order^27^; (3) Selective attention—assessed with Trail Making Test (TMT) B, which is as in Part A, the patient draws lines to connect the circles in an ascending pattern, but with the added task of alternating between the numbers and letters^27^; (4) Executive function—measured by Category Fluency Test (Animal).^28^, ^29^ Subjects were asked to produce words about animal as many as they could within 1 minute. The total number of correct responses positively reflects the test performance. (5) Visual perception—measured with Benton Visual Retention Test.^30^ It consists of 16 items. A set of target stimuli with four sets of stimuluses are shown in each item. Only one set of stimuli is matched with the target stimuli. The subject was required to identify which one of the four stimuluses was matched with the target stimuli. Two marks are given to each correct response. Thirty‐two marks is the maximum score.; (6) Simple auditory attention—assessed with Forward Digit Span Test, which is subtest of the Wechsler Intelligence Scale for Children‐Third Edition (WISC‐III).^31^ A series of digits were read to the subject and they were asked to repeat the numbers in the same order.; (7) Auditory working memory—assessed with Backward Digit Span Test.^32^ Subjects would listen to a series of numbers and they were asked to repeat them in a reverse order in the test. The length of the digits would be increased after every correct response in both tests.

All tests above have been validated in Chinese population and there are ranges for cognitive performance levels (Superior/Normal/Impaired).^26^, ^29^, ^33^ MS patients were defined as cognitively impaired (CI) when having impairment on three or more domains of above cognitive tests. Otherwise were defined as cognitively preserved (CP).

### Statistical analysis

SPSS version 23.0 was used for statistical analysis. All values were reported as mean (standard deviation) or median (range) as appropriate. Comparisons of demographic features, volumetric metrics and clinical scores between groups were carried out using *t*‐test, Mann–Whitney *U*‐tests and Pearson’s chi‐square test as appropriate. We used binary logistic regression to identify predictors of cognitive impairment. In the regression model, age, gender and educational years were first entered in block 1. The additional explained variance of MRI measures—NWMV, NGMV, normalized thalamic volume, WML load and WM integrity—were examined by entering separately in block 2. The DTI metric which showed the strongest association with cognitive outcome was included as a measure of WM microstructural integrity due to the dependency of MD and FA.

## Results

### Participants

A total of 36 RRMS patients were recruited in the study. All patients underwent clinical assessment and structural MRI, including 3‐dimentional T1‐weighted and FLAIR sequences. The mean interval between cognitive assessment and MRI scan is 2.7 +/‐ 2.1 months. Thirty‐four (94%) patients were successfully processed by volBrain and analyzed. Twenty‐nine (81%) patients acquired DTI (5 patients refused additional scan with DTI sequence) and were all successfully processed and analyzed. The demographic details and clinical features of CI patients and CP patients are displayed in Table 1. Fifteen (42%) patients were classified as CI and twenty‐one (58%) were classified as CP. There was no significant difference between CI and CP patients for age, gender, educational years, disease duration, number of relapses, and EDSS score.

**Table 1:** Main demographics and clinical features of cognitive impaired (CI) and cognitive preserved (CP) patients.

	Cognitive impaired (CI) N = 15	Cognitive Preserved (CP) N = 21	*P*‐value
Female sex, n (%)	11 (73%)	17 (81%)	0.679
Age, years, mean (SD)	35.8 (8.2)	32.6 (9.7)	0.32
Educational, years, mean (SD)	13.8 (3.5)	14.6 (2.8)	0.469
Disease duration, years, mean (SD)	8.1 (5.2)	7.0 (5.2)	0.67
No. of Relapses, median (range)*	2 (0‐4)	3 (0‐9)	0.438
EDSS score, median (range)	1.5 (0‐3.5)	1.0 (0‐6.5)	0.388

Pearson’s chi‐square test was used to test difference in gender, whereas unpaired t‐test/Mann‐Whitney test was used to test all other measures. EDSS, Expanded Disability Status Scale. Superscript in table: * The number of clinical relapses before the MRI, not including the first onset.

### Neuropsychological profile

**Figure 1:**
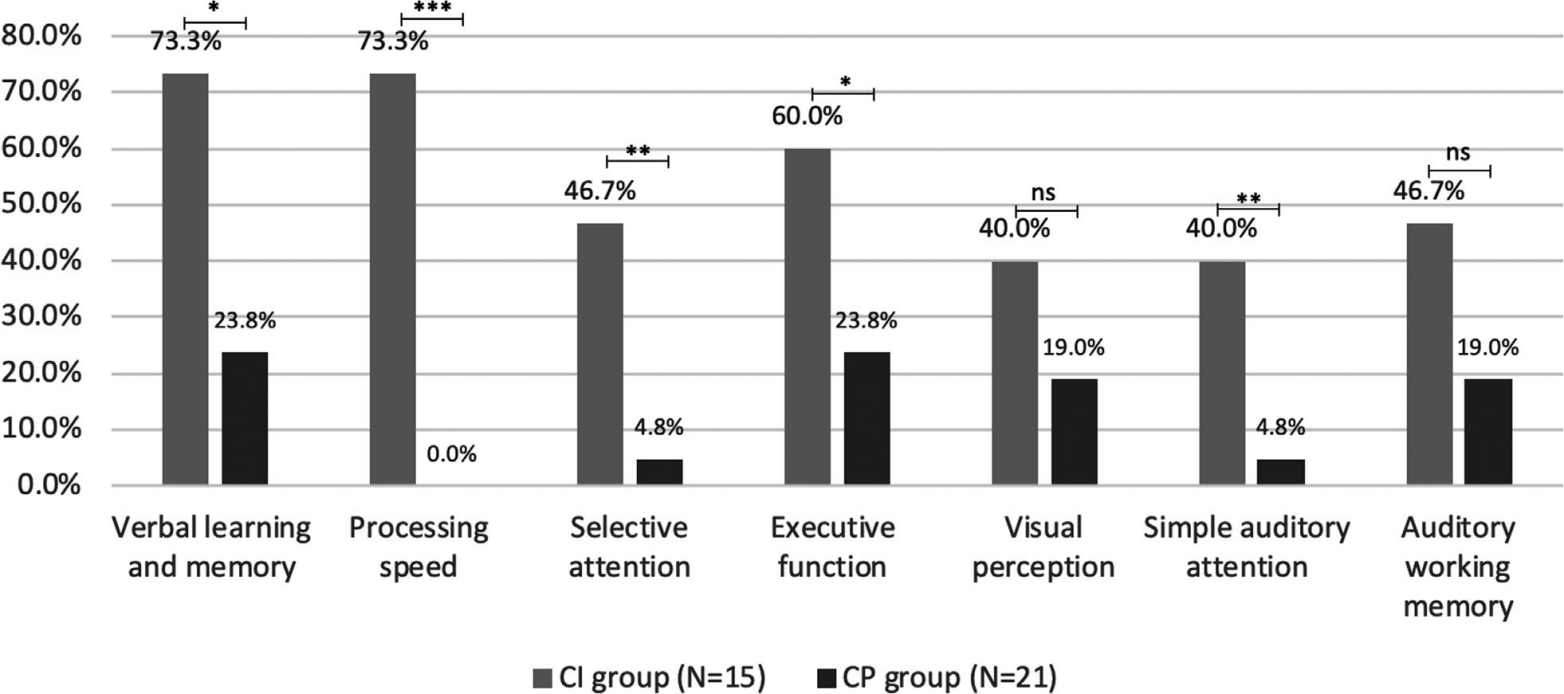
Cognitive profile in 36 RRMS patients in cognitively impaired group and cognitively preserved group. The bars indicated proportion of a specific cognitive function impaired. Pearson’s chi‐square test was used to test significant difference of a specific impaired cognitive domain.

All 36 patients completed neuropsychological testing. Overall, verbal learning and memory was the most commonly impaired domain (n = 16, 44%), followed by executive function (n = 14, 39%), processing speed (n = 11, 31%), auditory working memory (n = 11, 31%), visual perception (n = 10, 28%), selective attention (n = 8, 22%) and simple auditory attention (n = 7, 19%). Only 8 (22%) patients were completely free of cognitive domain deficit, while 13 (36%) and 15 (42%) patients had 1‐2 and more than 2 impaired domains, respectively. When comparing the proportion of patients with specific domain impairment, higher proportion of patients in CI group had impairment in almost all domains, except visual perception and simple auditory attention (Figure 1). When comparing cognitive scores between CI patients and CP patients, CI patients performed worse in processing speed (0.23 vs. 0.64, *P* < 0.001), simple auditory attention (−0.69 vs. 0.06, *P* < 0.05) and selective attention (0.1 vs. 0.9, *P* < 0.001).

### MRI Characteristics

#### White matter lesion (WML) burden

**Figure 2:**
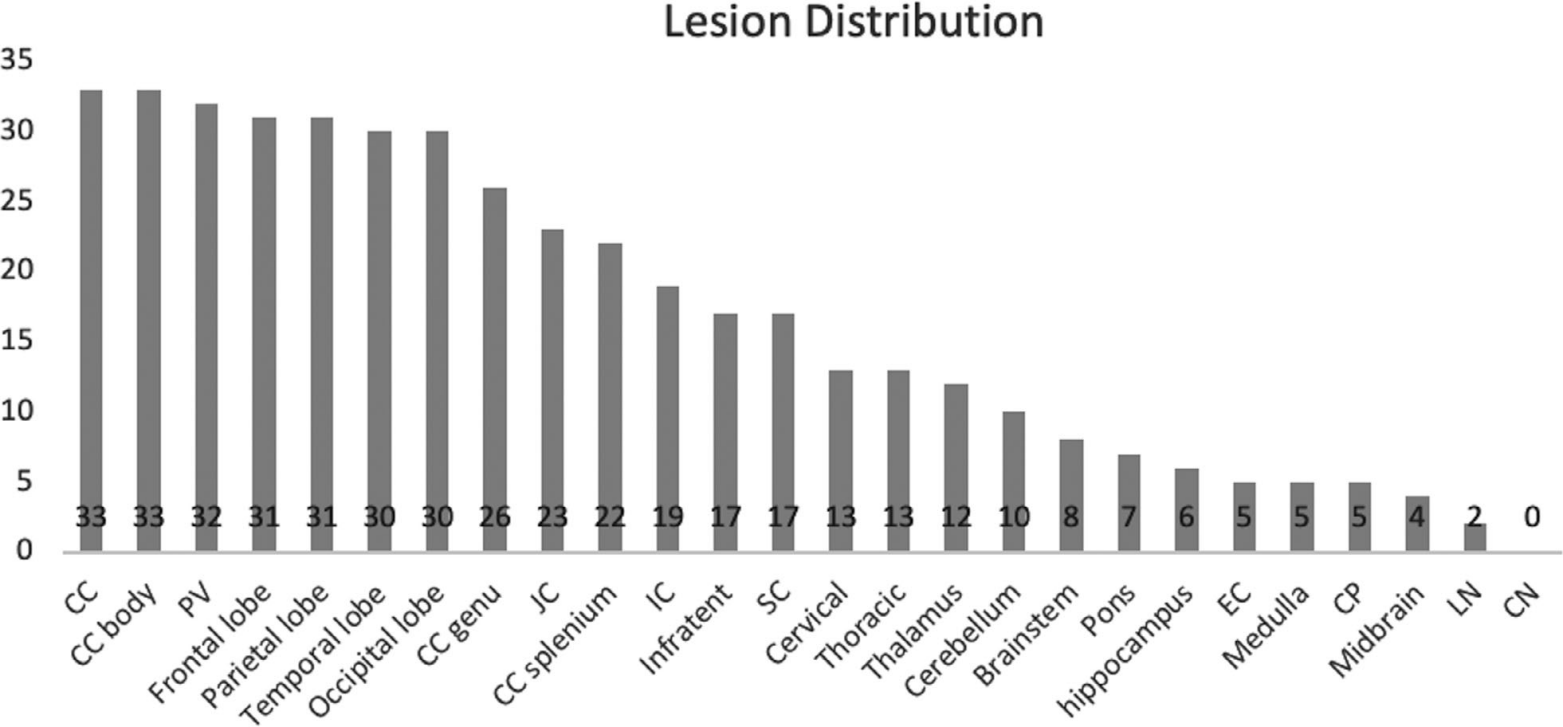
Lesion distribution in the study cohort of 36 RRMS subjects. The number on each bar represented number of patients with lesions in the specific anatomical region. CC, corpus callosum; PV, periventricular area; JC, juxtacortical area; IC, internal capsule; SC, spinal cord; EC, external capsule; CP, cerebellar peduncle; LN, lentiform nucleus; CN; caudate nucleus.

Distribution of WML on brain MRI is shown in Figure 2. Overall, the corpus callosum (CC) is the most commonly affected structure (n = 33, 92%), and the body is most susceptible among CC subregions. Almost half of patients (n = 17, 47%) have infratentorial lesions. Among subcortical structures, thalamus (n = 12, 33%) is the most commonly affected site. However, there is no significant difference in distribution of WML between CI and CP patients.

#### Whole‐brain MRI measures and global DTI measures

Similarly, the normalized global and regional brain volumes (Table 2), did not reveal significant differences between the two groups. Mean skeleton FA value was more abnormal in CI patients than CP patients (275.45 vs. 283.61 × 10^−3^, *P* = 0.023). CI and CP patients did not differ with respect to mean skeleton MD value.

**Table 2:** MRI characteristics between cognitive impaired (CI) and cognitive preserved (CP) patients. The only significant MRI parameter that differs between CI patients and CP patients is mean skeleton FA value.

MRI measures	CI	CP	p‐value
Normalized volume (mL)^a^			
Whole brain	80.02 (4.21)	82.19 (3.56)	n.s
White matter	30.94 (3.76)	32.90 (2.55)	n.s
Gray matter	49.08 (2.07)	49.30 (2.17)	n.s
Putamen	0.58 (0.07)	0.59 (0.09)	n.s
Thalamus	0.67 (0.11)	0.74 (0.13)	n.s
Globus pallidus	0.17 (0.03)	0.17 (0.02)	n.s
Hippocampus	0.56 (0.05)	0.55 (0.07)	n.s
Amygdala	0.12 (0.02)	0.12 (0.02)	n.s
Accumbens	0.04 (0.01)	0.04 (0.01)	n.s
Caudate	0.51 (0.05)	0.50 (0.05)	n.s
Global DTI measures^b^			
Global FA (×10^−3^)	275.45 (10.34)	283.61 (7.89)	**0.023**
Global MD (×10^−6^ mm^2^/s)	396.27 (17.25)	385.83 (7.82)	n.s
Lesion volume (mm^3^)^c^	17.87 (17.11)	10.72 (11.61)	n.s

Mean (SD) was reported. Unpaired t‐test/Mann‐Whitney test was used to test all measures. Significant values are shown in bold. FA, fractional anisotropy; MD, mean diffusivity; n.s, not significant. Superscript in table: a: sample size in analysis n = 34; b: sample size in analysis n = 29; c: sample size in analysis n = 36.

#### Predictors of cognition

Mean skeleton MD (×10^−6^ mm^2^/s) was selected as the measure of WM integrity, because it showed a significant association with cognitive impairment with an OR 1.14 (95% CI: 1.006 to 1.293, *P* = 0.041), with age, gender, and educational years adjusted. The logistic regression model including mean skeleton MD explained 43.6% (Nagelkerke R^2^) of the variance in presence of cognitive impairment. Increasing mean skeleton MD was associated with increased likelihood of developing cognitive impairment (β = 0.131). The model including mean skeleton FA explained 37.5% (Nagelkerke R^2^) of the variance in presence of cognitive impairment with an OR 0.875 (95% CI: 0.775 to 0.988, *P* = 0.031). Decreasing mean skeleton FA value was associated with increased likelihood of exhibiting cognitive impairment (β = −0.134). The regression model with only age (β = 0.269, *P* = 0.569), gender (β = −0.146, *P* = 0.654) and educational years (β = −0.9, *P* = 0.494) only explained 7.9% of variance of impaired cognition in RRMS patients. Including other MRI measures to regression model containing only demographical factors led to a small increase of explained variance: 7.8% by including NWMV (β = −0.230, *P* = 0.226), 1.2% by including NGMV (β = −0.140, *P* = 0.619), 2.8% by including normalized thalamic volume (β = −1.399, *P* = 0.547) and 2.7% by including WML load (β = −0.051, *P* = 0.475).

#### Extent of microstructural abnormalities: comparing CI and CP patients by TBSS

**Figure 3:**
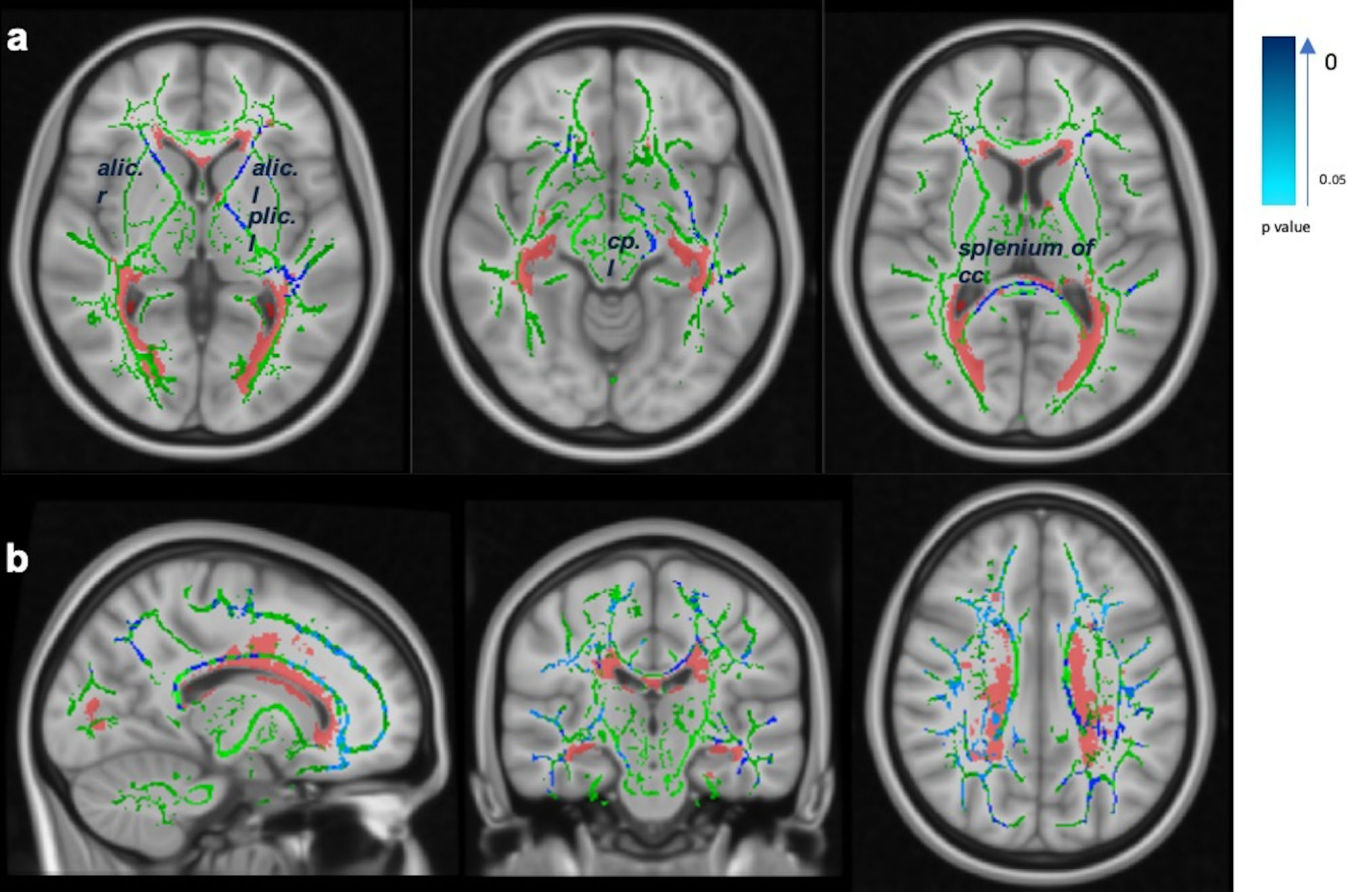
TBSS results are shown in blue on mean FA skeleton (green) and MNI 1mm standard space, overlaid by LPM (red). Tracts in deep blue displayed smaller p value or more significant results. (a) Reduced mean skeleton FA value mainly located on splenium of corpus callosum, left anterior and posterior limb of internal capsule and left cerebellar peduncle (Family‐wise error corrected p value less than 5% was reported) in CI patients comparing to CP patients (b) Widespread areas of increased mean skeleton MD in CI patients compared with CP patients. Two sample t‐test, FEW corrected and adjusted for age, gender and educational years. TBSS, Tract‐Based Spatial Statistics; LPM, lesion probability map; cc, corpus callosum; alic, anterior limb of internal capsule; plic, posterior limb of internal capsule; cp, cerebellar peduncle; l, left; r, right.

Specifically, CI patients presented significant reduction of mean skeleton FA values in left internal capsule, splenium of corpus callosum and left cerebellar peduncle, which was rarely overlapping with the LPM (Fig. 3a). We observed similar regions for increased mean skeleton MD values. Additionally, the right inferior longitudinal fasciculus, the right inferior fronto‐occipital fasciculus and the right corona radiata (posterior and anterior) were the most extensively affected tracts in CI patients relative to CP patients. In contrast, we only observed increase of MD in body of corpus callosum overlapped with lesioned area (Fig. 3b).

#### Correlations between extent of microstructural abnormalities and cognitive performance by TBSS

**Figure 4:**
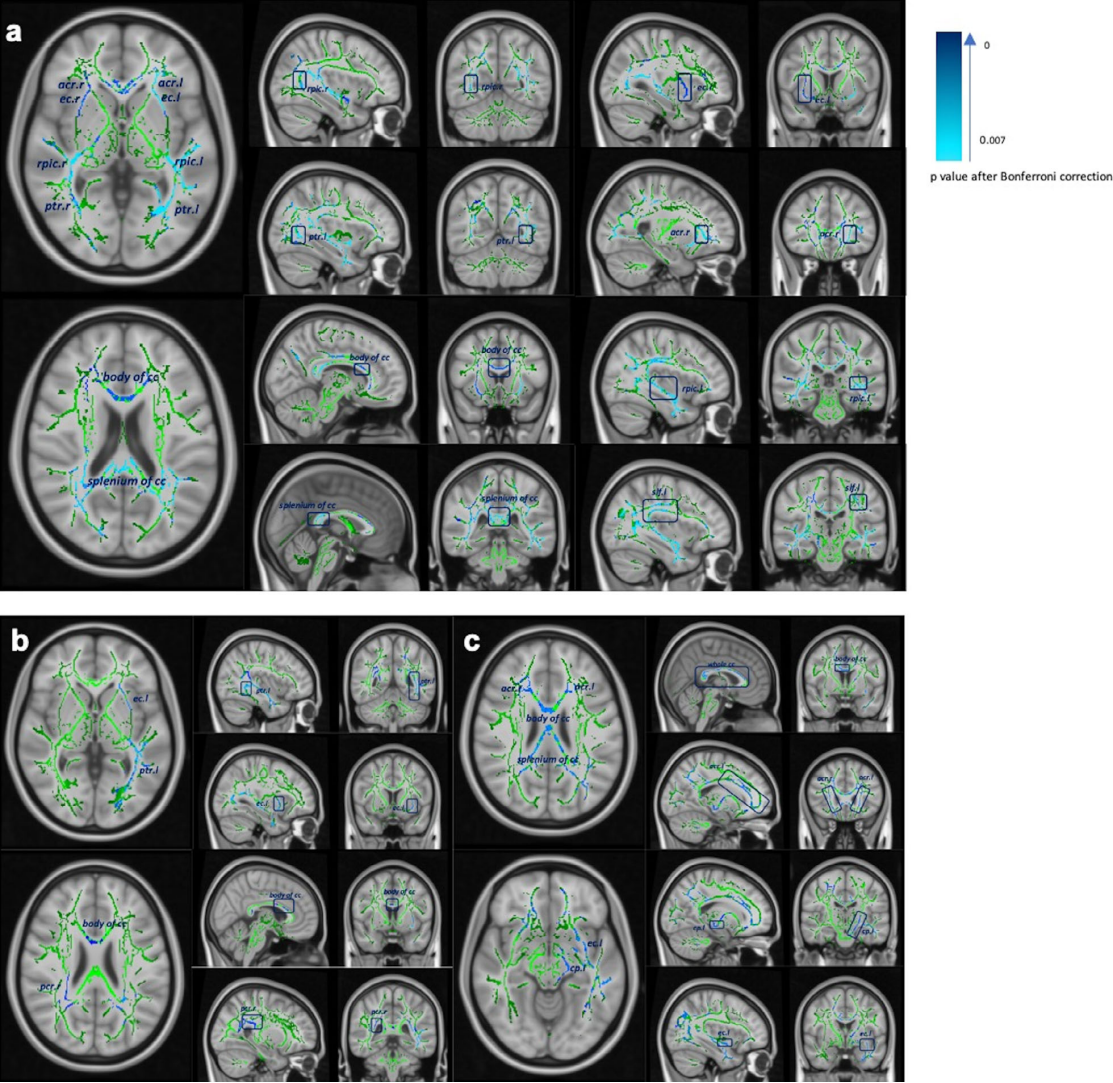
TBSS results are shown in blue on mean FA skeleton (green) and MNI 1mm standard space. Tracts in deep blue displayed smaller p value or more significant results. (a) Areas where positive correlation between FA and verbal learning and memory performance (higher score, worse performance) (b) Areas where positive correlation between FA and processing speed performance (higher score, worse performance) (c) Areas where positive correlation between FA and selective attention performance (higher score, worse performance). Correlation analysis, in all 29 patients with FWE corrected, *P* < 0.007 after Bonferroni correction adjusted for age, gender and educational years. TBSS, Tract‐Based Spatial statistics; cc, corpus callosum; rpic, retrolenticular part of internal capsule; ptr, posterior thalamic radiation; ec, external capsule; acr, anterior coronal radiate slf, superior longitudinal fasciculus; acr, anterior coronal radiate; cp, cerebellar peduncle; l, left; r, right.

In all patients, verbal learning and memory was positively correlated with mean skeleton FA value in corpus callosum, bilateral internal capsule, bilateral external capsule and bilateral posterior thalamic radiation. (*P* < 0.007, with FWE and Bonferroni correction) (Fig. 4a). A positive correlation was also observed between processing speed and mean skeleton FA value in body of CC, left external capsule and left posterior thalamic radiation (*P* < 0.007) (Fig. 4b). Mean skeleton FA value in corpus collosum, bilateral anterior corona radiata, and left cerebellar peduncle was positively correlated with selective attention after FWE and Bonferroni correction (*P* < 0.007, with FWE and Bonferroni correction) (Fig. 4c).

For mean skeleton MD value, significant negative correlations were noted between verbal learning and memory and similar tracts after FWE and Bonferroni correction (*P* < 0.007). However, the corpus collosum, right anterior coronal radiate represented significant negative correlation with selective attention (*P* < 0.05), which did not survive to Bonferroni correction.

## Discussion

In this study, we evaluated a cohort of Chinese RRMS patients with structural MRI and DTI and investigated the associations with cognitive impairment. Our data reinforced that loss of microstructural integrity is critical to cognitive impairment in Chinese RRMS patients. Demographic factors, whole‐brain MRI measures and WML load did not contribute to our prediction model for cognitive impairment. DTI, which could reflect microstructural integrity combining with TBSS, showed its importance and clinical relevance in further understanding cognitive dysfunction in MS.

We observed a slightly higher proportion of patients (up to 78%) with deficits on at least one cognitive domains in our cohort, as compared with previous studies (40‐70%).^1^ More importantly, we found that the most severely affected domains were verbal learning and memory and executive function, rather than processing speed that is most commonly reported in other studies.^34^, ^35^ This could be related to the difference in genetic and educational background of studied population, and possibly the difference in neuropsychological battery used.

The key strength of our study is the emphasis on microstructural MRI parameters and the relationship with a specific cognitive domain. We did not find significant differences in the WML burden, whole‐brain MRI measures between CI and CP patients. On the other hand, using DTI, we noted a mildly lower mean skeleton FA value in CI patients, which is consistent with previous studies suggesting that mildly lower mean skeleton FA are found in patients with cognitive impairment due to myelin damage.^9^, ^35^, ^36^ Microstructural integrity significantly explained 43.6% of the variance of cognitive impairment in this RRMS cohort, together with age, gender and educational years. Mean skeleton MD was more extensively affected in CI patients than mean skeleton FA, which supports that mean skeleton MD adds more effect than mean skeleton FA to the regression model to predict the presence of cognitive impairment.

At a regional level of microstructural integrity, abnormalities of several cognition‐relevant WM tracts—corpus callosum, left internal capsule, bilateral posterior thalamic radiation, right cerebellar peduncle, the right inferior longitudinal fasciculus, the right inferior fronto‐occipital fasciculus and the right corona radiata (posterior and anterior)—significantly differed between CI and CP patients. Specifically, the splenium of CC was prominently affected in our cohort. Furthermore, the microstructural abnormalities in CC were related to deficits of verbal learning and memory, processing speed, and selective attention performance. CC is the largest compact white matter fiber bundle of the human brain involved in interhemispheric transfer, and has been found a close relationship with cognitive deficits in multiple sclerosis.^13^ We also noted that cerebellar peduncle on the left side is affected and correlates with cognitive performance, which supports the hypothesis that cognition is affected due to disconnection between cerebellar nuclei and thalamus.^37^ Another finding is the correlation between posterior thalamic radiation and verbal learning and memory performance, which is in agreement with the hypothesis that posterior thalamic radiation is associated with cognitive performance.^38^ Fibers within posterior thalamic radiation project to occipital, temporal, and parietal cortex, and are thereby connected to cortical regions involved in the processing of the body image.^39^

The changes in DTI measures between CI and CP patients were unlikely to be primarily due to WML. We found that very few white matter tracts overlapped with WML distribution, suggesting that the loss of microstructural integrity only partially depends on primary damage of WML on strategic white matter tracts. This finding could be explained by secondary degeneration due to disruption by demyelination on a given white matter tract; but this hypothesis would be best addressed by longitudinal DTI studies.^40^ On the other hand, consistent with other studies, our data suggest that WML load assessment alone may not be adequate to assess and monitor for cognitive impairment in MS, whereas damaged fibers in normal appearing white matter may induce the cognitive deficits.^1^

Our study enrolled patients with an average disease duration of 7.5 years and showed discrepancies between change of microstructural integrity and whole‐brain MRI measures. Given the reasonable disease duration and observation period, we believe that our data is consistent with the hypothesis that loss of microstructural integrity may occur before brain tissue atrophy, and that DTI is likely a more sensitive biomarker for early cognitive impairment.

Regarding the limitations of our study, firstly, the small sample size and lack of healthy controls may limit the generalizability of our findings. Secondly, we did not account for cortical lesions in our analysis, which has also been reported to predict worsening cognition.^41^ Thirdly, since our study is cross‐sectional by design, we were not able to demonstrate the trajectories of evolution of WM and GM damage and their reciprocal interactions in a prospective manner. Large‐size prospective studies with healthy controls and detection of cortical lesions are warranted to further validate our findings.

## Conclusion

Our study confirmed that verbal learning and memory performance is preferentially affected in Chinese RRMS patients. Loss of microstructural integrity is more relevant to cognitive impairment than whole‐brain MRI measures and white matter lesion load in RRMS.

## Conflict of Interests

No.
